# Evidence of changes in the oral language in children born full-term and small for gestational age: a systematic review

**DOI:** 10.1590/1984-0462/2022/40/2021049IN

**Published:** 2022-05-11

**Authors:** Noemi Vieira de Freitas Rios, Luciene da Cruz Fernandes, Caio Leônidas Oliveira de Andrade, Luan Paulo Franco Magalhães, Ana Cecília Santiago, Crésio de Aragão Dantas Alves

**Affiliations:** a Universidade do Estado da Bahia, Salvador, BA, Brazil.; b Universidade Federal da Bahia, Salvador, BA, Brazil.

**Keywords:** Infant, low birth weight, Infant, small for gestational age, Child language, Speech, Recém-nascido de baixo peso, Recém-nascido pequeno para a idade gestacional, Linguagem infantil, Fala

## Abstract

**Objective:**

To perform a systematic review in order to verify the association between full-term birth of small for gestational age (SGA) children and the outcomes in the development of oral language.

Data source:

Articles from MEDLINE/PubMed, Web of Science, Embase, Lilacs, SciELO and Cochrane Library databases were identified, selected and critically evaluated by two independent reviewers and a judge, blindly, without language restriction and publication period. The PRISMA tool was used, and original studies with a theme involving children born full-term and SGA were included, outcome related to aspects of oral language development, as well as the use of tests, scales and/or specific questionnaires for the investigation, whose methodology was described in full, with children as the target population.

Data synthesis:

The researchers included nine articles based on the eligibility criteria. Studies have shown that being born SGA can interfere in aspects related to language and reported greater chances of under performance in SGA children when compared to children with appropriate size for gestational age. It was observed that the different studies did not have a uniform design, and the objectives were quite diverse. Furthermore, few of them had as focus issues related to the assessment of language, as well as the variability of instruments used to investigate this domain.

**Conclusions:**

The effects of low weight for gestation age in full-term infants continue beyond the neonatal period and may impact on children’s performance, mainly with regard to oral language development.

## Resumo

**Objetivo:**

Realizar uma revisão sistemática para verificar a associação entre o nascimento a termo de crianças pequenas para a idade gestacional (PIG) e os desfechos no desenvolvimento da linguagem oral.

**Fontes de dados:**

Artigos dos bancos de dados MEDLINE/PubMed, Web of Science, Embase, LILACS, SciELO e Cochrane Library foram identificados, selecionados e avaliados criticamente por dois revisores independentes e um juiz, às cegas, sem restrições de idioma e período de publicação. A ferramenta PRISMA foi utilizada e foram incluídos estudos originais envolvendo crianças nascidas a termo e PIG, desfechos relacionados a aspectos do desenvolvimento da linguagem oral, bem como o uso de testes, escalas e/ou questionários específicos para a investigação, cuja metodologia estava descrita na íntegra, com crianças como população-alvo.

**Síntese dos dados:**

Nove artigos foram incluídos a partir dos critérios de elegibilidade. Os estudos demonstraram que nascer PIG pode interferir em aspectos relacionados à linguagem e relataram que as chances de crianças PIG apresentarem um desempenho inferior são maiores quando comparadas as com tamanho adequado para a idade gestacional. Observou-se que os diferentes estudos não tinham um delineamento uniforme e seus objetivos eram bastante diversificados. Além disso, poucos focavam em questões relacionadas à avaliação da linguagem e foi possível notar uma variabilidade de instrumentos utilizados para investigar esse domínio.

**Conclusões:**

Os efeitos do baixo peso ao nascer em nascidos a termo persistem além do período neonatal e podem ter impacto no desempenho infantil, principalmente no que se refere ao desenvolvimento da linguagem oral.

## INTRODUCTION

Proper development of the individual depends on factors related to pre-, peri- and postnatal life. Birth weight and ideal gestational age are considered prerequisites for favoring this development and, in addition to other biological and environmental factors, directly influence the future quality of life of the children.^1,2^ Therefore, a careful look at children born full-term and small for gestational age (SGA) is necessary.

SGA children are those with birth weight below the expected for the respective gestational age in weeks. Therefore, it is more often considered as the weight below the 10^th^ percentile, based on the intrauterine growth curve,^3–5^ and may be associated with intrauterine growth restriction (IUGR). Full-term are the ones born with gestational age between 37 and 41 weeks and 6 days, and when the weight is less than 2,500g, in addition to SGA, the literature classify them as underweight.^6,7,8^

Studies have shown that the prevalence of live births with low weight can vary from 4 to 15%,^9,10^ with 3 to 4% full-term. In Brazil, this rate is 4.3%, which means more than 7 thousand children/year,^11^ a high number that must be considered in the elaboration of public policies and health promotion and prevention actions.

The causes of SGA birth and the risk factors related to it vary,^12,13^ from genetic to environmental factors.^14^ Children born SGA have a higher risk of developing diseases such as obesity, coronary heart disease, high blood pressure, type 2 diabetes mellitus, dyslipidemia, delayed neuropsychomotor development, and visual, auditory, behavioral and learning problems,^15,16,17,18,19^ which directly interfere in the process of the speech and language development.^20,21,22^ Therefore, the relationship between complications during this process and the low weight of children born full-term needs more research to find more conclusions.

In previous reviews,^20,23^ studies took into account low weight and prematurity. In the present article, the authors assessed the low weight of children born full-term classified as SGA and hypothesized that a large part of full-term children and SGA are susceptible to presenting changes in the development of language skills.

However, this is not a topic with a comprehensive approach in the specialized literature, whose research has diversified designs. Moreover, data on these skills are little explored and often inconclusive, generating important questions regarding the dimension of the altered linguistic aspects.

Therefore, this investigation focused on verifying findings in the literature that specify the association between the term birth of SGA children and the outcomes in the development of oral language.

## METHOD

The present investigation considered the PICO structure^24^ to describe the components related to the identified problem and to structure the following research question: Is there evidence that changes in oral language may be present in SGA children born full-term?

The selection of articles was executed in the electronic databases independently and blindly, using the Mesh descriptors previously defined. Each reviewer, separately, judged the inclusion of articles based on reading the titles and, when available, the abstracts. After the first stage, the articles were selected and thus they were read in full to confirm eligibility and inclusion in the study. In a third moment, the results found were confronted among two reviewers, and the disagreements were resolved through a judge (third reviewer), with expertise in systematic review.

The researchers included original studies with a theme involving children born full-term and SGA, regardless of the reference curve used. These studies needed to have some outcome related to the occurrence of problems in the development of oral language. Furthermore, the use of tests, scales and/or questionnaires specific to the investigation were also included in this article. Studies whose methodology was fully described, with a target population of children (aged between two and ten years), without language restrictions and period of publication, with texts available in full, were prioritized.

The researchers excluded duplicate articles, literature review, case reports or case series, studies that evaluated aspects of children’s language exclusively in children born with low weight and premature, or that did not relate the gestational age of the evaluated SGA population.

The systematic literature review was conducted according to the Preferred Reporting Items for Systematic Reviews and Meta-Analyzes (PRISMA) methodology.^25^ The selection of the studies was made based on the titles found initially and, after being selected, the abstracts were read. In cases in which reading the abstract was not sufficient to establish whether the article should be incorporated, the text was read in full to determine its eligibility. When abstracts were sufficient, full versions were selected to confirm eligibility and inclusion in the study.

The search strategy was based on the electronic databases: MEDLINE/PubMed, Web of Science, Embase, Lilacs, SciELO and Cochrane Library. The articles were identified between July 2019 and January 2020. The descriptors used as a search strategy were: low birth weight, LBW, Small for Gestational Age, SGA, language, speech, Speech-Language Pathology, combined through Boolean operators OR and/or AND, resulting in the search details as follows: “low birth weight” <OR> “LBW” <OR> “Small for Gestational Age” <OR> “SGA” AND language * OR speech * OR speech language pathology.

The criteria proposed by the Effective Public Health Practice Project (EPHPP — Quality Assessment Tool for Quantitative Studies)^26,27^ were used in order to assess the quality of the evidence and methodological of the studies, especially the details regarding the selection bias, study design, potential confounders, blindness of researchers and participants, data collection methods (if they were valid and reliable), losses follow-up (exclusion or loss of follow-up), integrity of the intervention and appropriate analysis of the research question. According to the definition of each of these criteria, the studies were then classified as poor quality (presence of two or more weak items), moderate (presence of only one weak item) or strong (absence of weak items).

As for the quality of scientific writing, the Strengthening the Reporting of Observational Studies in Epidemiology (STROBE)^28^ elaboration guide was adopted in order to verify the accuracy of scientific writing in the selected studies. The quality index of each article corresponded to the sum of the total number of items assessed as positive, with the maximum score being 22 (100%). Articles with 50% or more agreed with criteria considered to be of regular quality and those with more than 75%, of good quality.

The analysis of the studies found was performed in a descriptive manner and executed in three stages. The first included the description of the following methodological characteristics: authorship, country/year of publication, study design, objective, sample, test or protocol used to assess aspects of language. The second stage comprised the analysis of the outcome and the factors associated with it, that is, research instruments, main results with description of aspects of the target audience’s language, conclusion and EPHPP and STROBE score. The third step consisted of analyzing the aspects of language addressed in each text, considering the linguistic subsystems pragmatic, morphosyntactic, lexical and phonological and other cognitive aspects. The results were presented as a narrative synthesis of the existing literature that relates the term SGA birth and aspects of children’s language.

## RESULTS

**Figure 1.**
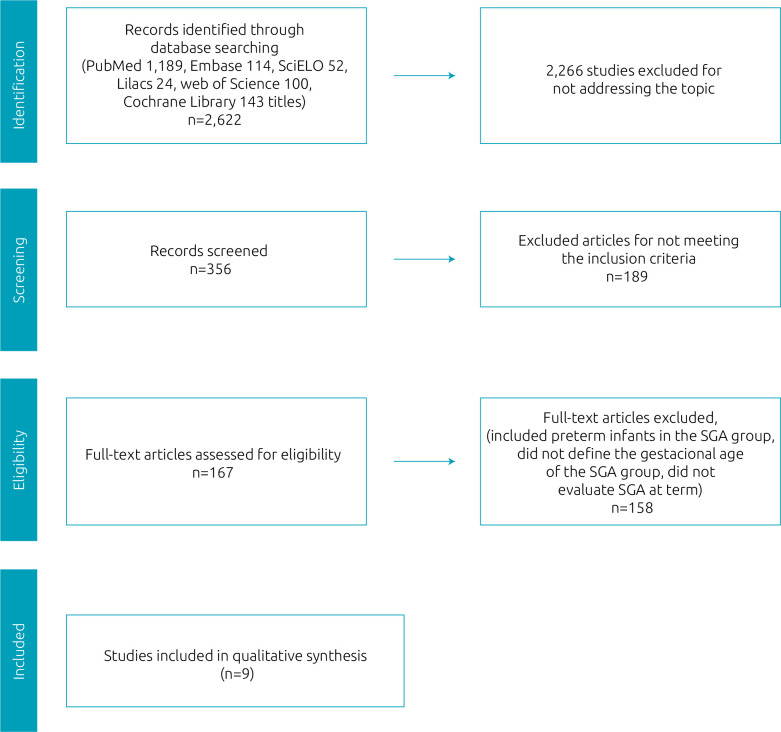
Flowchart of article selection based on PRISMA criteria.

Figure 1 illustrates the selection process of the articles that composed the present review. Of the total, 2,613 articles were excluded because they did not meet the required inclusion criteria; however, nine articles were included based on the eligibility criteria.

As shown in Table 1 of the selected studies, one was done in Brazil^29^ and eight were published internationally. The sample number of studies ranged from 10^29^ to 3,738^30^ full-term SGA children.

**Table 1.** General characteristics of the studies included in the systematic review.

Author, country, year	Study design	Sample characteristics
Castro Conde et al, Spain, 2019^37^	Prospective cohort	50 SGA, 54% boys, P: 2140.14±330.09, GA: 37.73±1.73 44 AGA, 47.70% boys, P: 2987.16±522.32, GA: 38.1±2.0 Age in the evaluation: 2 years
Takeuchi et al., Japan, 2018^31^	Population-based/ longitudinal	581 SGA, P: 3074±379 495 SGA with Catch Up, 38% boys, GA: 39.1 86 SGA (15%) without Catch Up, 51.2% boys, GA: 38.7 31952 AGA, 51.8% boys, P: UNS, GA:39.1 Age in the evaluation: 2,5 years
Takeuchi et al., Japan, 2016^30^	Longitudinal	3738 SGA, 52.4% boys, P: UNS, GA: 38.7, 42825 AGA, 51.9% boys, P: UNS, GA: 38.9 Age in the evaluation: 2.5 years
O’Neill et al., Ireland, 2016^32^	Prospective cohort	51 SGA, 54% boys, P: 2850±255 GA: 39.64±1.3 51 TGA, 32% boys, P: 3215±394, GA: 39.53±1.40 13 STGA, P and GA UNS 189 AGA, 47% boys, P: 3567±422, GA: 39.81±1.20 Age in the evaluation: 2–2.6 years
Simões et al., Spain, 2015^33^	Cohort, longitudinal	33 SGA, 69.70% boys, P: 2304±263, GA: 38.1±0.9 26 AGA, 57.7% boys, P: 3375±403, GA:39.8±1.4 Age in the evaluation:2 years
Savchev et al., Spain, 2013^34^	Consecutive cohort	112 SGA, 54.5% boys, P:2416±280, GA:38.8±1.2 111AGA, 55.9% boys, P:3396±370, GA :39.8±1.1 Age in the evaluation: 2–2.2 years
Klarić et al., Croatia, 2012^35^	Cross-section of a longitudinal cohort, case-control	50 SGA with IUGR, 44% boys, P: UNS GA: 277d 50 AGA, 44% boys, P: UNS, GA: 279d Average age at evaluation: 6 years and 4 months
Walker et al, Jamaica, 2010^36^	Case-control study of a longitudinal cohort	109 SGA (IG: 55, P: 2190±200, GA: 38.5±0.9 and CG: 54, P: 2240±180, GA: 38.6±0.9), 44.4% boys 73 AGA, 47.9% boys, P: 3130±330, GA: 39.4±0.8 Age in the evaluation: 6 years
Oliveira et al., Brazil, 2003^29^	Analytical case-control study of a longitudinal cohort	10 TNB/SGA, 20% boys, P: 2323±127, GA :273.7 d 10 PTNB/AGA, 50% boys, P:2262±174, GA:253.2d 47 TNB/AGA, P:UNS, GA: UNS Age in the evaluation: 6, 12 and 18 months.

SGA: small for gestational age; AGA: appropriate for gestational age; GA: gestational age; P: average birthweight; Catch Up: weight gain in height; TGA: thin-for-gestational age; STGA: small and thin-for-gestational age; UNS: unspecified; IUGR: intrauterine growth restriction; IG: intervention group; CG: control group; TNB/SGA: newborns full-term and small for their age gestational age; PTNB/AGA: preterm newborns and adequate birth weight for gestational age; TNB/AGA: term newborns and adequate weight for gestational age.

Regarding the SGA classification, a Japanese study considered birth weight below the tenth percentile for gestational age (GA) and birth length below -2.0 standard deviation (SD) for GA, or birth weight below -2.0 SD for GA and length below the tenth percentile for GA.^31^ In the other studies^29,30,32–37^ only birth weight below the tenth percentile for GA was considered according to local standards, based on the uterine growth curve.^3,5^ Some authors^30^ reported that they disregarded height due to inaccuracies in the measurements at birth, following the evidence.^4^

It was found that only one article was classified as having poor quality of evidence, while the others were considered to have moderate to strong evidence according to the EPHPP criteria. The article considered weak also had a small sample size.^29^ As for the quality of scientific writing, one article was considered of regular quality^29^ and the others were classified as of good quality (Table 2).

**Table 2.** Characteristics of the studies included in the systematic review of changes in aspects of oral language of children born full-term small for gestational age.

Author	Findings/language	STROBE	EPPHP
Castro Conde et al.^37^	SGA lowest score on the Bayley scale. In the language domain, they presented an average of: SGA: 95.3 (88.91–101.69), AGA: 108.61 (100.72–116.50), p<0.010	87.1%	Strong
Takeuchi et al.^31^	SGA without Catch Up are more likely to demonstrate developmental delays in all the behaviors examined. At 2.5 years, they are more likely to be unable to compose 2-word sentences (OR 3.58; CI95% 1.81–7.08), compared to AGA	78.4%	Strong
Takeuchi et al.^30^	SGA were more likely to fail climbing stairs and composing a two-word sentence at 2.5 years old (OR 1.5; CI95% 1.2–1.8) compared to AGA	75.2%	Moderate
O’Neill et al.^32^	In the language domain, it presented an average of: SGA: 109 (97–117), p=0.570, AGA: 109 (100–115), TGA: 100 (94–109), p=0.024, had significantly lower scores in the three domains, with a reduction of 0.35 SD in language	80.9%	Strong
Simões et al.^33^	SGA lowest score on the Bayley scale, compared to AGA language domain averages: SGA: 95.4±15.1 and AGA: 108.1±19.2	78.3%	Strong
Savchev et al.^34^	SGA lowest score on the Bayley scale. In the language domain, average SGA: 94.7±14.8, AGA: 101.0±16.5, p=0.025. SGA risk of low language scores, even after adjusting for potential confounders.	77.8%	Strong
Klarić et al.^35^	SGA with IUGR presented worse language results compared to the AGA group. There were statistically significant differences (p<0.001) in language comprehension, total expressive language (vocabulary, structure, content), naming skills and repetition of words without meaning	82.5%	Strong
Walker et al.^36^	SGA in the CG had poorer selective attention and visuospatial memory, but there were no differences in IQ language	81.7%	Strong
Oliveira et al.^29^	6m — performance expected for age 9m — delay in babbling expression 12m — statistically significant delay in the TNB/SGA group, which remained with polysyllabic babbling 18m — delay persisted in an infant in the TNB/SGA group	55.8%	Weak

SGA: small for gestational age; AGA: appropriate for gestational age; TGA: thin-for-gestational age; UNS: unspecified; IUGR: intrauterine growth restriction; IG: intervention group; CG: control group; TNB/SGA: newborns full-term and small for their age gestational age; CI95%: 95% confidence interval; OR: *Odd Ratio*; IQ: intelligence quotient; SD: standard deviation.

In the reviewed studies, there was a diversity of objectives, with few being directed to the evaluation of some aspect of language.^29,35^ In the other selected studies,^30–34,36,37^ this ability was described as being part of the tests that assessed cognitive skills and neurobehavioral aspects of development.

Table 3 shows that the Bayley scale was the instrument used in four analyzed studies. It was used the average age group of two years to perform the assessment.^32–34,37^ In other studies,^30,31^ aspects of language were mentioned, but they used questionnaires directed at parents who investigated issues related to neurodevelopment and behavior as instruments.

**Table 3.** Description of the research instruments, and their respective methods of application, in studies that assessed language skills.

Authors	Data collection instrument	Cognitive-linguistic subsystems evaluated and test limitations
Takeuchi et al.^30,31^	Questionnaire with questions consistent with Denver-II	Questions divided into three categories (motor development, language development and social and personal development) that the child already reaches at 2.5 years old. The inability to perform each behavior at 2.5 years of age was defined as developmental delay. The three questions in the language category: Can your child say words with meaning? Can your child compose two-word sentences? Can your child say his own name? Test limitations: Did not use instruments to assess linguistic and behavioral aspects of children.
Castro Conde et al.,^37^ O’Neill et al.,^32^ Simões et al.,^33^ Savchev et al.^34^	Bayley Scale BSID-III	Subdivided into 5 domains: Cognition, Language (expressive and receptive communication), Motor (thick and thin), Social-emotional and Adaptive Component. In the study,^32^ the first 3 domains were considered. Test limitations: The scale assesses children from 1 to 42 months. The screened aspects of language are not explored in the results, it only mentions the total score of the child obtained on the language scale, which is justified because it is not the objective of the scale to provide isolated parameters of the evaluated domains, but rather the profile of neurodevelopment that encompasses all five domains.
Klarić et al.^3^5	Reynel’s language development scale and other tests such as Naming test, Mottier test, Cuturic development test	The following skills were analyzed: expressive language, verbal comprehension. Vocabulary, Structure and Content of the language, Nomination, Time for naming in seconds, Mottier Test, Development quotient evaluated by the Cuturic test. Test limitations: Despite the use of many tests, the article does not describe the results of the skills assessed.
Walker et al.^3^6	WPPSI-III, PPVT, digit sequence, Corci test blocks, daily attention test, test for reading evaluation, SDQ	The article evaluated: IQ, receptive vocabulary, Short-term auditory memory, visuospatial memory, attention, reading and behavior (emotional symptoms, conduct problems, hyperactivity and relationships with others). Test limitations: As the article proposed to evaluate other parameters, such as reading ability, the other aspects of language were not addressed, only expressive vocabulary.
Oliveira et al.^29^	ELM Scale	It is a scale applicable to children from 0 to 36 months of age, performed quickly, with direct testing of the child or with questions addressed to the parents. It assists in determining patterns of linguistic behavior expected for each stage of child development. It comprises the expressive auditory, auditory-receptive and visual areas. Test limitations: Use of a single tool to address language levels in development.

WPPSI-III: Wechsler Preschool and Primary Scale of Intelligence, 3rd edition; PPVT: Pea-body Picture Vocabulary Test; SDQ: Strengths and Difficulties Questionnaire; IQ: intelligence quotient; ELM: Early Language Milestone Scale.

In the study^35^ it was confirmed the use of tests that contemplated various aspects of language. Authors^36^ carried out a research in children with LBW, born full-term, to verify whether psychosocial stimulation, up to the age of 2 years, benefited the development of cognition and behavioral aspects at 6 years of age. The sample of SGA children was divided into an intervention group, which received stimulation for two years, and another control group, which did not receive it, and compared it with appropriate size for gestational age (AGA) children. The receptive vocabulary was assessed using the PPVT image test (Pea-body Picture Vocabulary Test), the only skill analyzed in the study related directly to aspects of language. Other aspects, such as memory, attention, reading ability and behavior, were also evaluated.

It was observed that the received intervention contributed for the group to present better performances in the aspects evaluated at 6 years of age, however there were no significant differences mainly in the assessment of linguistic ability.

Table 4 shows the aspects of language that were possibly analyzed in each study. These aspects were selected from the analysis of the instrument or technique applied in the studies. It was noted that data related to semantics and morphosyntax were the most analyzed in the reviewed texts.

**Table 4.** Aspects of children’s language analyzed in the included studies.

Linguistic aspects	Selected studies									Total (%)
	Castro Conde et al.^37^	Takeuchi et al.^31^	Takeuchi et al.^30^	O’Neill et al.^32^	Simões et al.^33^	Savche et al.^34^	Klarić et al.^35^	Walker et al.^36^	Oliveira et al.^29^	
Pragmatic	+	–	–	+	+	+	+	–	–	55.5
Phonology	+	–	–	+	+	+	+	–	–	55,5
Semantics	+	+	+	+	+	+	+	+	+	100.0
Morphosyntactic	+	+	+	+	+	+	+	–	+	88.8
Other cognitive aspects*	+	–	–	+	+	+	+	+	–	66.6

*Note: aspects related to cognition on the Bayley scale, Intelligence quotient, attention and memory.

## DISCUSSION

According to what was observed, the studies showed that SGA birth can interfere in aspects related to language and reported higher chances of underperformance in SGA children when compared to AGA children.

These results, however, must be interpreted with caution, since the different studies did not have a uniform design, the objectives were quite diverse and few had as focus issues related to the assessment of linguistic skills, in addition to the variability of instruments used to investigate that domain.

It was found that studies with full-term SGA children are not frequent, especially when it relates to aspects of development including language. Studies with this population have important limitations, such as different assessment instruments; small, heterogeneous and sometimes non-representative samples of the population; precarious detailing of clinical and sociodemographic characteristics, among others. For this reason, the two studies^30,31^ that used a questionnaire directed to parents were not excluded.

Even considering the absence of child evaluations as a limitation, preventing more targeted conclusions, these studies^30,31^ arouse the scientific environment for the investigation of outcomes related to the development of speech and language since at least two questions of the instruments used were about linguistic aspects. Screenings, as those used by the authors, may serve to identify children at risk for aspects of neurodevelopment, even though it is subjective because it is based on their parents’ opinions. Therefore, this perception is of fundamental importance and often contributes to the early diagnosis process.

The findings revealed that SGA birth was a risk factor for developmental delay among children who were born full-term, corroborating with other studies.^4,33,34^ The authors^30,31^ highlighted the importance of continuous monitoring, in order to detect behavioral problems and provide appropriate interventions to SGA children, especially those with failed growth speed (catch up). The authors of this review questioned the other aspects of language that were not addressed and the lack of more precise conclusions related to the development of speech and language.

It is noteworthy that most of the texts analyzed and included in this review related the SGA birth to other clinical states and their effects on child development, among them, some linguistic aspects. It was noticed that language was one of the aspects evaluated within cognitive skills, being analyzed specifically in a few studies.^29,35^

Linguistic aspects were related because it is part of children’s neurobehavioral development. Authors^38,39,40^ already mentioned the importance of evaluating these aspects throughout development. The Bayley III scale was the instrument used in four studies.

The use of the scale makes it possible to identify and quantify developmental delay, but longitudinal assessments of the child are essential to complete any type of change and enable the necessary referrals for therapeutic interventions to minimize future side-effects.

In the three studies in this review that used the scale,^33,34,37^ a lower performance was found in the assessed domains in the SGA group when compared to the AGA group, as it was not observed in the study by O’Neill et al.^32^

When children born full-term SGA were evaluated at 2 years of age, authors also obtained lower scores in the mentioned domains of the scale.^34^ In the study, the result of neurodevelopment at 2 years of term SGA newborns with and without Doppler changes in the umbilical artery was evaluated, ruling out placental dysfunction. Even without initially presenting a placental dysfunction that justifies SGA birth, the study highlighted that babies are in need of a more differentiated look at development, as there is evidence of delays and may be suggestive of interrupted neurological maturation during pregnancy.

The SGA children that were evaluated in a study^37^ in which the objective was to quantify the rates of immature neonatal electroencephalogram (EEG) patterns and associate them with neurodevelopment were diagnosed with IUGR between the second and third trimester by Doppler and biometric measurements on fetal ultrasound, and confirmed with birth weight <tenth percentile. The authors found a significant correlation between interhemispheric asymmetry and lower scores on motor and language development.

IUGR appeared as one of the factors associated with SGA birth. Authors^4,41,42,43,44^ revealed that not all SGA babies are pathologically small and there are several reasons for SGA birth, such as gestational age at birth, ethnicity, parents’ stature, presence of fetal abnormalities, fetal exposure to alcohol or drugs, and maternal diseases. Studies^4,19,45^ showed that full-term SGA children with or without IUGR had lower scores in the formal assessment of neurodevelopment. It is believed that, since the language is an intrinsic skill and dependent on these aspects, it is possible to present atypical development in full-term SGA children.

Two revised texts^35,37^ evaluated SGA children with IUGR. The results showed that children with IUGR had worse language results compared to the control group. These difficulties were present in the comprehension of language, content, structure and in the reduced size of the vocabulary that compromises the comprehension and expression of the language. These processes occur in frontotemporal areas; therefore, they suggest that any compromise in the volume and structure of this area can contribute to difficulties in understanding.

Asymmetric IUGR affects the frontal cortex neural networks, with direct implications for learning and memory functions,^46,47^ and, as suggested by authors,^48^ impairments also in auditory processing that directly interferes with speech perception.

In this study, it was observed that the children presented lower results in the phonological coding and decoding tests, which are important for the process of learning to read and write. It is understood that losses in these skills may imply difficulties mainly with reading.^49,50^ The authors^35^ conclude that IUGR has a negative impact on language development, which is evident in pre-school age. As a limitation of the study, the authors pointed out the difficulty of differentiating babies who actually had IUGR and those who were just SGA for other reasons.

It is observed that semantics and morphosyntax are the language skills most analyzed in the studies in this review. In five of these studies,^32–35,37^ some scales were used that generally contemplate all aspects of oral language, in addition to other skills related to development. In view of the scientific and methodological quality of the five texts reviewed, it is believed that these results are efficient, showing lower performance of the SGA group in the linguistic domains evaluated when compared to the AGA group, except in one study.^32^ In the other texts that included semantic and morphosyntactic aspects, in at least two of them,^29,30^ the data were little explored and methodological flaws were observed, however they pointed out that being born full-term and SGA are risk factors for possible language development delays.

It was found that pragmatics and phonology were the language skills with the lowest percentage of analysis. It is understood that the understanding of what is spoken anticipates the expression. The pragmatic and semantic subsystems are the first to be observed in child development. The communicative exchanges supported by vocal, verbal and non-verbal means reveal the limited linguistic capacity, preventing the correct production of the sounds of the language and the structuring of more complex phrases.^51,52^

The conventional use of the oral language develops with the appearance of the first words, followed by the production of simple phrases, followed by the complex ones, until reaching proficiency and becoming a native speaker, with the phonological system completely acquired. This process is complex and it involves several factors that can directly interfere with the child’s linguistic performance, such as neurocognitive, auditory-perceptual, linguistic, individual, interactional, environmental and sociocultural.^53,54^

Some authors^55^ cited instruments used in the assessment of the spoken language of premature preschoolers. It was found that, of the eight, six were international instruments, most of them development scales. Besides that, Brazilian researchers built two of them. It is believed that there is a lack of tests considered gold standard with methodological and scientific rigor, not only in Brazil but also in other countries.

It is noticed that the scores of the normative processes for the population that applies, when they exist, were obtained through validation with unrepresentative numbers and reduced numbers of subjects. The importance of studies in this area is emphasized, using the most appropriate methodology, covering all linguistic aspects, and therefore producing more consistent data.

It can be concluded that the effects of low weight continue beyond the neonatal period and can have an impact on the child’s performance, especially in regard with issues related to language.

This finding is of great relevance for the competent bodies to implement public policies aimed to this population, such as early diagnosis and intervention programs, in addition to drawing the attention of health professionals and family members who must remain alert to any changes in development. The language assessment of children born full-term and SGA, as well as the monitoring in the early and school phases, can prevent future learning problems and favor aspects related to mental health by avoiding losses in the development of speech and language, which ends up being a high cost problem for the individual and society.

An important political issue in our country, considering resource constraints, is to decide whether interventions in child development should only be for children considered at risk, and this mainly relates to prematurity, or also, for those considered to be SGA, as the evidence of this review underline the need to reach children with low weight, including those born full-term. There is an important gap related to this topic and its intention is to encourage future research.

In view of the possibilities of changes in the development of children’s language in this population, the scarcity of studies that assess linguistic skills and monitor development, as well as the lack of interest on the part of the existing literature, to delve deeper into the investigations of such aspects, are emphasized once again. Futhermore, some limitations need to be considered. The lack of standardization in the tests and the reduced number of subjects, added to the heterogeneity of the tests and analysis of the results, made interpretation difficult, as well as the generalization of the results, and made it impossible to perform meta-analyzes. Another limitation was that the researched gestational age, which restricted the number of selected articles. The vast majority of articles researched related to low weight, focused on studying prematurity.

It is essential to properly assess the development of children born low weight and full-term, since they are also susceptible to changes in development and these are more prevalent in relation to those who were born suitable for gestational age.

In conclusion, the nine articles selected from the eligibility criteria pointed out that being born full-term and SGA can interfere in aspects related to language. The effects of low weight on SGA and full-term children continue beyond the neonatal period and can have an impact on children’s performance, especially in regard to issues related to the development of oral language.
